# Diagnostic Accuracy in Detecting Fungal Infection with Ultra-Low-Dose Computed Tomography (ULD-CT) Using Filtered Back Projection (FBP) Technique in Immunocompromised Patients

**DOI:** 10.3390/jcm13061704

**Published:** 2024-03-15

**Authors:** Luigia D’Errico, Anita Ghali, Mini Pakkal, Micheal McInnis, Hatem Mehrez, Andre C. Schuh, John G. Kuruvilla, Mark Minden, Narinder S. Paul

**Affiliations:** 1 Department of Medical Imaging, Toronto General Hospital, 200 Elizabeth St., Toronto, ON M5G 2C4, Canadaanita.ghali@liverpoolft.nhs.uk (A.G.); mini.pakkal@uhn.ca (M.P.);; 2 Department of Radiology, Royal Papworth Hospital NHS Foundation Trust, Papworth Road, Cambridge Biomedical Campus, Cambridge CB2 0AY, UK; 3 Department of Radiology, Aintree University Hospital, Liverpool University Hospitals NHS Foundation Trust, Lower Lane, Merseyside, Liverpool L9 7AL, UK; 4 Canon Medical Systems Canada Limited, 75 Tiverton Court, Markham, ON L3R 4M8, Canada; 5 Princess Margaret Cancer Centre, 610 University Ave, Toronto, ON M5G 2C1, Canada; andre.schuh@uhn.ca (A.C.S.); john.kuruvilla@uhn.ca (J.G.K.); mark.minden@uhn.ca (M.M.); 6 Department of Medical Imaging, London Health Sciences Centre and St. Joseph’s Hospital, Western University, 339 Windermere Road, London, ON N6A 5A6, Canada

**Keywords:** chest CT, ultra-low dose, immunocompromised

## Abstract

**Purpose:** To compare the accuracy of ultra-low-dose (uLDCT) to standard-of-care low-dose chest CT (LDCT) in the detection of fungal infection in immunocompromised (IC) patients. **Method and Materials:** One hundred IC patients had paired chest CT scans performed with LDCT followed by uLDCT. The images were independently reviewed by three chest radiologists who assessed the image quality (IQ), diagnostic confidence, and detection of major (macro nodules, halo sign, cavitation, consolidation) and minor (4–10 mm nodules, ground-glass opacity) criteria for fungal disease using a five-point Likert score. Discrepant findings were adjudicated by a fourth chest radiologist. Box–whisker plots were used to analyze IQ and diagnostic confidence. Inter-rater reliability was assessed using interclass correlation coefficients (ICCs). The statistical difference between LDCT and uLDCT results was assessed using Wilcoxon paired test. **Results:** Lung reconstructions had IQ and diagnostic confidence scores (mean ± std) of 4.52 ± 0.47 and 4.63 ± 0.51 for LDCT and 3.85 ± 0.77 and 4.01 ± 0.88 for uLDCT. The images were clinically acceptable except for uLDCT in obese patients (BMI ≥ 30 kg/m^2^), which had an IQ ranking from poor to excellent (scores 1 to 5). The accuracy in detecting major and minor radiological findings with uLDCT was 96% and 84% for all the patients. The inter-rater agreements were either moderate, good, or excellent, with ICC values of 0.51–0.96. There was no significant statistical difference between the uLDCT and LDCT ICC values (*p* = 0.25). The effective dose for uLDCT was one quarter that of LDCT (CTDI_vol_ = 0.9 mGy vs. 3.7 mGy). **Conclusions:** Thoracic uLDCT, at a 75% dose reduction, can replace LDCT for the detection of fungal disease in IC patients with BMI < 30.0 kg/m^2^.

## 1. Introduction

Opportunistic fungal infections in immunocompromised (IC) patients are associated with a mortality of 38–92% [1,2,3,4]. Early diagnosis is challenging, particularly for invasive fungal infection (IFI). In patients where histopathological correlation is difficult, a diagnosis of probable or possible IFI can be made using a combination of host factors, positive cultures, and specific radiologic findings on thoracic imaging [5].

A common approach to imaging IC patients with fever or respiratory symptoms is to assess pulmonary involvement using chest radiography (CXR), start empirical broad-spectrum antibiotics early, and add antifungal agents if fever or symptoms persist, while awaiting the results of non-invasive tests [6,7]. With severe immunodeficiency, the systemic and pulmonary inflammatory response to infection may be poor or virtually absent. CXR may be normal or show minimal non-specific abnormalities in IC patients with underlying lung infection. Opportunistic infections such as PCP pneumonia present with ground-glass densities that are easily missed with chest X-ray [8,9]. Standard helical chest CT has more utility than CXR in IC patients, especially in those with AIDS [10] and hematological malignancies [11]; both techniques use ionizing radiation but chest CT is performed at a relatively high effective patient dose of ~5 mSv [12].

Thoracic low-dose CT (LDCT) provides a useful alternative to CXR and standard-dose thoracic CT (SDCT). LDCT, performed at an effective patient dose of ~1–2 mSv, has superior utility for early diagnosis of pulmonary infection in IC patients compared to CXR [6,7,13], and is performed at 20–30% of the radiation dose of SDCT. Our regional cancer center uses LDCT as the standard of care in thoracic imaging for all IC patients with hematological malignancy prior to treatment and during clinical presentation with febrile neutropenia (FN).

It is not unusual for IC patients to have frequent thoracic LDCT scans during a hospital admission that can last several weeks, with CT used to monitor response to therapy [14]. Repeated CT scans lead to cumulative radiation exposure [15] and increase the lifetime risk for developing a second malignancy [16]. Therefore, there is an urgent need to validate thoracic CT protocols that achieve diagnostic image quality using a sub-mSv effective patient radiation dose.

The primary goal of this study is to determine whether thoracic ultra-low-dose CT (uLDCT), performed using a 75% dose reduction compared to thoracic LDCT, provides comparable diagnostic image quality and accuracy for detection of major radiological features of fungal chest infection in IC patients. A secondary goal is to evaluate how accurately lung lesions are characterized on uLDCT compared to LDCT. Our hypothesis is that uLDCT has comparable diagnostic utility to LDCT in demonstrating major radiological findings suggestive of fungal infection in IC patients.

## 2. Material and Methods

### 2.1. Study Design and Patients

This single-center prospective study was approved by our Institutional Research Ethics Board (REB # 14-7420-CE). We prospectively recruited 100 IC patients who were referred for a clinically indicated thoracic LDCT.

### 2.2. Inclusion Criteria

Patients aged ≥18 years, with an established diagnosis of a hematological malignancy and a clinical suspicion of opportunistic chest infection, or who were under surveillance of known fungal infection were included. A study coordinator who was outside the patient’s immediate circle of care obtained consent from each patient to have paired helical LDCT and uLDCT performed.

### 2.3. Exclusion Criteria

Patients were excluded if they were unable or unwilling to provide written consent.

### 2.4. Scan Protocol

Paired LDCT and uLDCT scans were obtained on a 64-slice scanner (Aquilion64, Canon Medical Systems, Otawara, Japan) during the same inspiratory breath hold or during consecutive breath holds. Filtered back projection (FBP) was used to reconstruct all images. CT protocol parameters required identical tube kilovoltage (135 kVp), gantry rotation time (0.5 s), detector collimation (64 × 0.5 mm), pitch factor 1.35 (86.4 mm/s), reconstruction slice thickness/interval (3.0/2.4 mm), and lung reconstruction kernel (FC03, U02, Boost). The only difference between the two image acquisitions was the fixed applied tube current of 40 mA for LDCT (CTDI_vol_ = 3.7 mGy) and 10 mA for uLDCT (CTDI_vol_ = 0.9 mGy).

### 2.5. Image Review

All studies were anonymized, randomized, and independently reviewed by three thoracic radiologists with expertise in reading LDCT (MP, MM, and LD with 15, 8, and 4 years’ experience, respectively). Any discrepancies were settled by a 4th thoracic radiologist (NP with 20 years’ experience of reading LDCT).

All readers participated in a single calibration session by reviewing 10 training sets to ensure consistency in assessment of image quality, radiological findings, and nomenclature. The training images were not included in the trial. Following the calibration session, uLDCT images were reviewed by each radiologist independently, in 4 batches of 25 patients, with anonymized studies presented in random order. Following a 1–2-week washout period, LDCT images were reviewed by each radiologist independently, in 4 batches of 25 patients, with anonymized studies presented in random order.

A stand-alone clinical PACS workstation was used to review images and score the following: (1) CT image quality (IQ); (2) diagnostic confidence based on IQ, (3) detection of major and minor radiological features of fungal infection, based on the European Organization for Research and Treatment of Cancer (EORTC) consensus paper [5] and recent literature [17,18,19,20,21]. Questionnaire categories, features, and Likert scores used for the assessment are presented in Table 1.

### 2.6. Image Quality (IQ) Assessment

Patients were grouped by BMI (kg/m^2^) based on World Health Organization (WHO) classification: underweight ≤18.5, normal 18.5–24.9, overweight 25–29.9, and obese ≥30. Data were analyzed based on BMI stratification. Qualitative scores for IQ were individually recorded for the three readers, and then combined to generate an average score for each LDCT and uLDCT study. LDCT and uLDCT results were compared within each BMI group.

### 2.7. Diagnostic Confidence Assessment

Assessment of diagnostic confidence in LDCT and uLDCT exams was performed using a similar approach as IQ assessment, and data were analyzed accordingly.

### 2.8. EORTC Radiological Findings of Fungal Disease Assessment

Each reader recorded major and minor radiological findings of fungal disease for each study (Table 1). Major radiological findings were categorized as a macro nodule, halo sign, cavitation, or consolidation. Minor radiological findings were classified as either GGO or 4–10 mm nodules. Discrepant findings, defined as a lack of complete agreement between all three radiologists on the presence or absence of a major or minor radiological feature, required independent adjudication of the entire study by a 4th radiologist, blinded to the previous reads. uLDCT findings were compared to LDCT results, which were considered as ground truth. Inter-rater reliability was assessed for each finding for both LDCT and uLDCT.

## 3. Statistics

Box–whisker plots of IQ and diagnostic confidence scores for each BMI group were generated. The mean, first quartile, median, third quartile, and data range for each BMI group was determined. The Wilcoxon paired *t*-test was used to compare uLDCT to LDCT scores. True positive, false negative, false positive, true negative, sensitivity, specificity, and accuracy of uLDCT for detection of major and minor radiological findings were calculated on a per patient basis. Inter-rater reliability was evaluated for each finding for both LDCT and uLDCT scans. Inter-rater reliability analysis was performed based on interclass coefficient (ICC) calculation for each task using the cloud-based tool “AgreeStat Analytics” [22]. Agreement between readers was considered poor, moderate, good, and excellent for ICC values <0.5, 0.5–0.75, 0.75–0.9 and >0.9; respectively [23]. Statistical significance was determined for a threshold of *p* < 0.05.

## 4. Results

All the patients had an underlying hematological malignancy, including leukemia, myeloma, lymphoma, myelodysplastic syndromes, and aplastic anemia. The patient cohort demographics and radiation doses are summarized in Table 2. LDCT and uLDCT scans had CTDIvol = 3.7 mGy and 0.9 mGy, respectively.

### 4.1. Image Quality (IQ) Assessment

**Figure 1:**
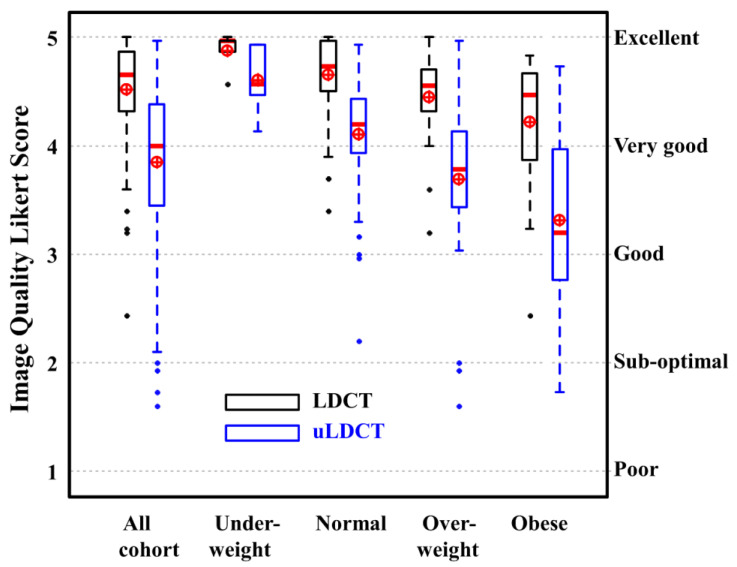
Box–whisker plots for averaged confidence visualization scores for anatomical features on lung reconstruction images for different BMI groups. Results for LDCT (LDCT) and ultra-LDCT (uLDCT) scores are presented in black and blue; respectively. Median and mean values are presented with red thick line and a circle with cross; respectively. Likert score values (1–5) are presented on the left y-axis and the corresponding image quality assessment (poor–excellent) on the right y-axis. The median [first quartile third quartile] scores for LDCT are 4.65 [4.33–4.87] (all cohort) 4.97 [4.87–4.97] (underweight), 4.73 [4.51–4.97] (normal), 4.55 [4.33–4.70] (overweight) and 4.47 [3.87–4.67] (obese). For uLDCT, the median [first quartile third quartile] scores are 4.00 [3.46–4.38] (all cohort), 4.57 [4.47–4.93] (underweight), 4.20 [3.94–4.43] (normal), 3.78 [3.43–4.08] (overweight) and 3.20 [2.77–3.97] (obese).

The IQ assessment reflected the readers’ confidence in identifying normal anatomical structures in the lungs. The box–whisker plots for the averaged IQ scores are presented in Figure 1. The averaged confidence visualization scores for all the LDCT images were higher than for all the uLDCT images (mean ± STD, 4.52 ± 0.47 vs. 3.85 ± 0.77; *p* < 0.05). However, on the subgroup analysis, there was no significant difference in the IQ between LDCT and uLDCT for the underweight patients (4.87 ± 0.18 vs. 4.61 ± 0.34, *p* = 0.13). The difference was statistically significant for the other BMI groups (*p* < 0.05). All the LDCT images had a clinically acceptable IQ (score > 3), except for one obese patient with BMI = 60.2 kg/m^2^. As demonstrated in Figure 1, the IQ scores for uLDCT in the normal and overweight patients were >3.0, indicating good–excellent confidence, except for five outliers. Although the median IQ scores for uLDCT in the obese patients were >3, there was a wide range that included suboptimal (2) and poor (1) scores.

**Figure 2:**
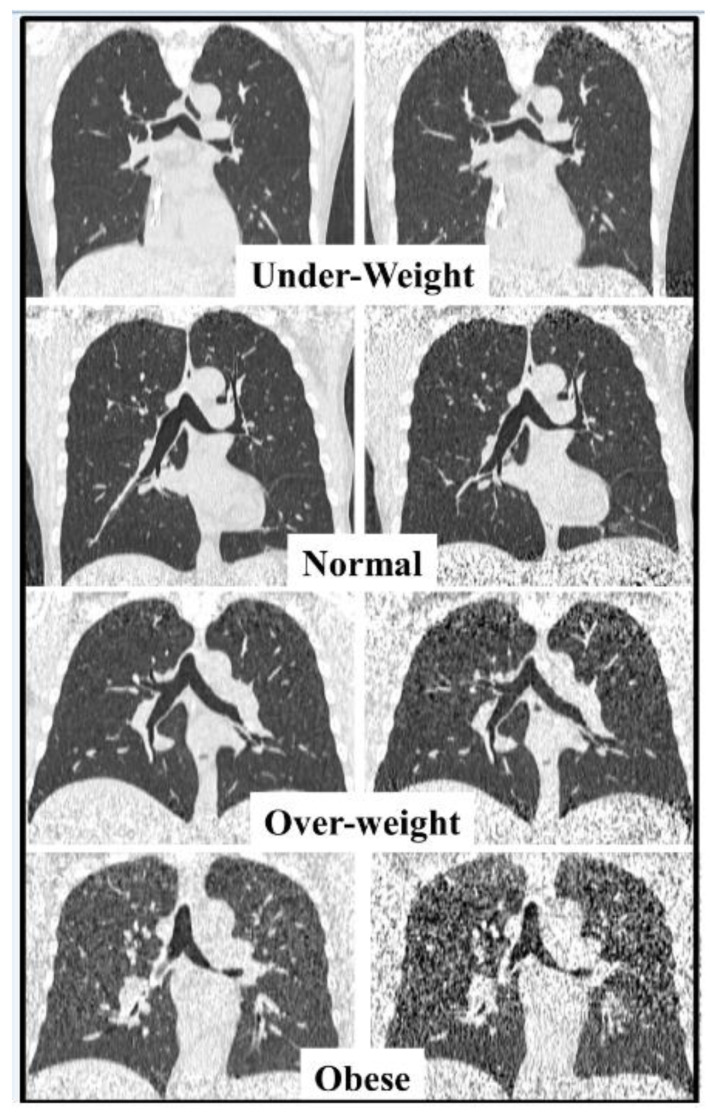
Four paired coronal CT images in patients scanned with LDCT protocol (**left column**) and ultra-LDCT protocol (**right column**). The ultra-LDCT image in the obese patient degrades substantially especially in the lung apex and close to the diaphragm due to photon starvation.

Figure 2 illustrates the reduction in image quality for uLDCT in the patients with increased BMI. This occurs due to the combined effects of a reduction in the number of x-ray photons produced during uLDCT compared to LDCT, and the increased absorption of these X-ray photons in patients with higher BMI.

### 4.2. Diagnostic Confidence Assessment

**Figure 3:**
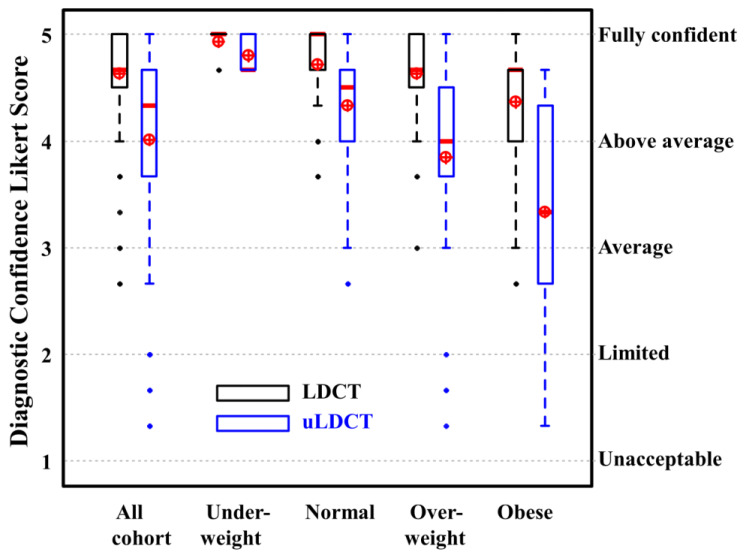
Box–whisker plots for diagnostic confidence score using lung reconstruction images for different BMI patient groups. Results for LDCT (LDCT) and ultra-LDCT (uLDCT) scores are presented in black and blue; respectively. Median and mean values are presented with red thick line and a circle with cross; respectively. Likert score values (1–5) are presented on the left y-axis and the corresponding diagnostic confidence assessment (unacceptable–fully confident) on the right y-axis. The median [first quartile third quartile] scores for LDCT are 4.67 [4.58–5.00] (all cohort), 5.00 [5.00–5.00] (underweight), 5.00 [4.67–5.00] (normal), 4.67 [4.58–5.00] (overweight), and 4.67 [4.00–4.67] (obese). For uLDCT, the median [first quartile third quartile] scores are 4.33 [3.67–4.67] (all cohort), 4.67 [4.67–5.00] (underweight), 4.50 [4.00–4.67] (normal), 4.00 [3.67–4.42] (overweight), and 3.33 [2.67–4.33] (obese).

The readers’ confidence in making a diagnosis of fungal infection on the LDCT and uLDCT scans are presented in Figure 3. The diagnostic confidence for the LDCT images was clinically acceptable (score > 3) in 99/100 (99%) patients; the single outlier had a BMI = 60.2 kg/m^2^. The averaged confidence visualization scores for all the LDCT scans were higher than for all the uLDCT scans (mean ± STD, 4.63 ± 0.51 vs. 4.01 ± 0.88; *p* < 0.05). There was no significant difference in the diagnostic confidence between the LDCT and uLDCT images for the underweight patients (4.93 ± 0.15 vs. 4.80 ± 0.18, *p* = 0.09). Although the difference was statistically significant for the other BMI groups (*p* < 0.05), the diagnostic confidence for uLDCT was clinically acceptable (score > 3.0) in 45/46 (98%) of the patients with a normal BMI, and in 25/28 (89%) of the overweight patients. Overall, the diagnostic confidence scores for uLDCT were clinically acceptable for the patients with BMI < 30 kg/m^2^, which represents 79/100 (79%) of our patient cohort. For the obese patients, although the median diagnostic confidence scores were >3, there was a wide range of scores including limited confidence (2) and unacceptable (1) scores.

### 4.3. EORTC Radiological Findings Assessment

Table 3 summarizes the results for the major and minor radiological findings of fungal infection. The uLDCT findings on a per patient basis were compared to the findings on LDCT. The sensitivity, specificity, and accuracy for any major radiological sign were 92.3%, 97.3%, and 96.0%, respectively. The corresponding values for any minor radiological sign were 84.5%, 81.3%, and 84.0%, respectively. The accuracy in detecting any subtype of major radiological finding was 95–100%. The sensitivity for uLDCT in scoring the halo sign was 66.7%, due to the mischaracterization of the halo sign in one out of three patients. For minor findings, ground-glass opacity (GGO) and 4–10 mm nodules, sensitivity, specificity, and accuracy were in the range of 77.8–89.3%.

**Figure 4:**
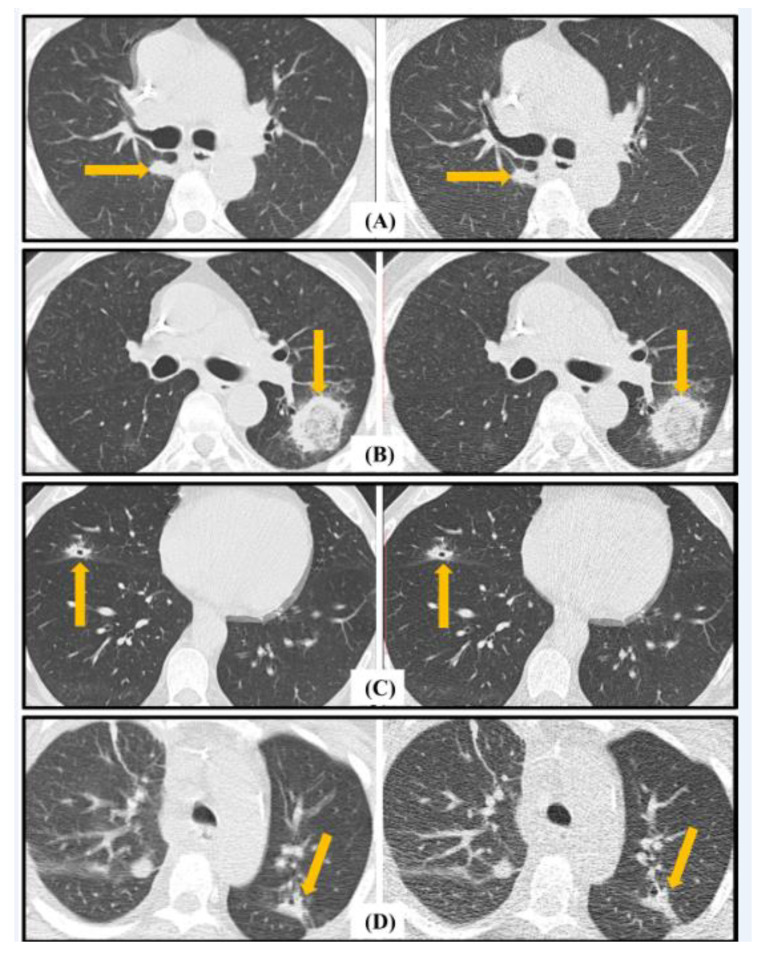
Major European Organization for Research and Treatment of Cancer (EORTC) radiological criteria for fungal disease demonstrated on LDCT (**left column**) and ultra-LDCT (**right column**). (**A**) demonstrates a macro nodule in the right lower lobe; (**B**) illustrates a halo sign in the superior segment of the left lower lobe; (**C**) demonstrates a cavitary nodule in the right middle lobe. (**D**) illustrates subsegmental consolidation in the left upper lobe. Pathological features are highlighted by arrows in each image.

**Figure 5:**
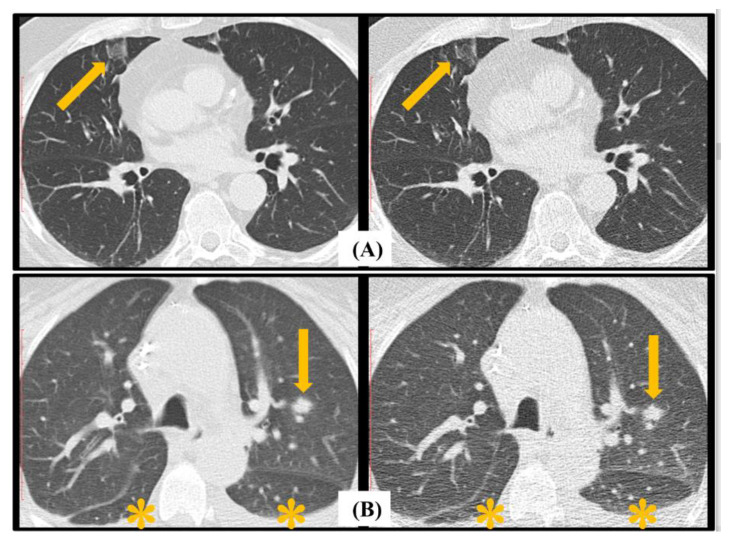
Minor European Organization for Research and Treatment of Cancer (EORTC) radiological criteria for fungal disease demonstrated on LDCT (**left column**) and ultra-LDCT (**right column**). Top row (**A**) demonstrates an 11 mm ground-glass opacity (GGO) in the medial segment of the middle lobe highlighted with an arrow. Bottom row (**B**) demonstrates a small central nodule in the left upper lobe, highlighted by an arrow, with bilateral pleural effusions highlighted by an asterisk (*). Both radiological findings are well demonstrated on uLDCT data despite increased background noise that results in significant reduction in lung parenchyma contrast.

The EORTC findings that are well depicted on LDCT and uLDCT are highlighted in Figure 4 and Figure 5. Despite a 75% reduction in the radiation dose resulting in increased noise for the uLDCT images, all the radiological features are well demonstrated. There was lack of agreement in the sub-categorization of the major radiological findings on uLDCT in six patients (three false negative and three false positive), as shown in Table 3. A direct comparison of the LDCT and uLDCT images performed after the initial reads were completed demonstrated that the discrepancy was due to a characterization error and not due to an error of detection.

The inter-rater reliability for LDCT and uLDCT data, assessed using interclass correlation (ICC), are displayed in Table 4. There was good inter-rater agreement for the detection of any major sign of infection and moderate inter-rater agreement for the detection of any minor sign of infection. There was moderate–excellent agreement for all subtypes of major findings and moderate agreement for minor radiological findings. The Wilcoxon paired *t*-test demonstrated no statistically significant difference in the ICC values between uLDCT and LDCT (*p* = 0.25).

## 5. Discussion

The diagnostic performance of uLDCT was compared to LDCT in 100 IC patients with underlying hematological malignancy. Our study demonstrated that uLDCT performed using a 75% radiation dose reduction compared to LDCT had a clinically acceptable image quality and diagnostic confidence in non-obese patients. There was no significant difference between uLDCT and LDCT in detecting major and minor radiological findings of infection. The inter-rater agreements for uLDCT were moderate to excellent and not significantly different from LDCT. These findings confirm that uLDCT should be considered for non-obese IC patients.

Twenty-five major radiological findings were detected on LDCT in the cohort of 100 patients. This is consistent with Escuissato et al. [17]. The accuracy of uLDCT in detecting major radiological findings was 95–100% with a moderate to excellent inter-rater agreement. There were three false negative and three false positive cases attributed to mischaracterization rather than detection errors. The accuracy in detecting the minor radiological criteria of GGO and 4–10 mm nodules with uLDCT was 79%-81%, with only moderate inter-rater agreements. While these values are smaller than for the major radiological findings, they fall within the clinical practice of the pulmonary detection of small parenchymal opacities with LDCT screening examinations [24,25]. These findings reflect the relatively small size of the target lung nodules (4–10 mm) and the reduced conspicuity of ground-glass opacities at the higher levels of image noise, consequent to the lower tube current used in uLDCT images.

Our results are consistent with Kim et al. [26], who achieved an acceptable diagnostic performance for pulmonary infection detection using uLDCT in febrile neutropenic patients with hematologic malignancy. Kim et al. found that most studies were clinically acceptable, and the three observers graded only one to four studies (out of 207) as unacceptable. The discrepancy with our study can be explained by differences in demographics; the highest BMI in their cohort was 29.0 kg/m^2^ whereas our cohort had 21 obese patients (BMI ≥ 30.0 kg/m^2^). A subgroup analysis of our results that excludes the obese patient cohort confirms that our results align with those of Kim et al., with only four studies deemed of below-average diagnostic quality. These results are promising given that the mean radiation dose used in their study was 27% higher compared to our study: 0.60 mSv vs. 0.43 mSv.

Gerritsen et al. [7] compared CXR to uLDCT in 67 febrile neutropenia patients and confirmed that the diagnostic utility of uLDCT was significantly superior to CXR. The mean radiation dose for uLDCT was 0.24 mSv, this was achieved using a tube voltage of 80 kVp. Although this provided a satisfactory diagnostic image quality for their patient cohort, the use of a low tube kilovoltage for non-contrast chest CT in adult patients is associated with significant image degradation, especially in patients with a higher BMI, and needs to be implemented cautiously. Ludes et al. [27] have confirmed superior image quality using 135 kVp compared to 80 kVp for uLDCT. It is likely that the level of diagnostic image quality in our study would have significantly decreased had we used a tube voltage of 80 kVp.

Laqmani et al. [28] evaluated the influence of iterative reconstruction (IR) on thoracic LDCT, CTDIvol = 1.7 mGy, in 30 IC patients, and compared the image quality to a separate cohort of 30 patients who had SDCT, CTDIvol = 7.6 mGy. The LDCT images were reconstructed with FBP and seven different IR levels. The LDCT cohort had a mean BMI of 23.9 kg/m^2^; a BMI range was not provided, so it is unclear whether obese patients were recruited. The image quality and lesion detection improved with LDCT-IR compared to LDCT-FBP with a radiation dose reduction of 78% from SDCT. These results demonstrate the value of IR algorithms in LDCT.

Nam et al. [29] evaluated the influence of deep learning reconstructions in ultra-low-dose chest CT of 100 patients, CTDIvol = 0.33 mGy. Five image series were reconstructed using a standard and sharp vendor-agnostic deep learning post-processing model (DLM); vendor-specific deep learning image reconstruction (DLIR, high), and adaptive statistical iterative reconstruction (ASiR, 70%) with standard and sharp settings. The vendor-agnostic deep learning algorithms provided the best image quality in terms of the image noise and spatial resolution. This is a promising approach to improving the image quality in uLDCT.

The present study has a few limitations. The principal limitation is that the CT images were reconstructed using filtered back projection without iterative or deep learning reconstructions. Iterative and deep learning reconstruction techniques demonstrate improvements in image quality for thoracic uLDCT, especially in obese patients. The routine clinical use of these algorithms in chest CT has increased within the last 5 years due to the availability of fourth-generation IR and new DLM algorithms. However, more than 64 per cent of the CT units currently in operation across Canada are six years old or older, and 34 per cent of CT units are older than ten years! [30,31]. This means that a substantial number of clinical CT units do not have access to iterative or deep learning reconstruction and rely on filtered back projection for clinical diagnosis. Therefore, the findings from this study are very relevant to current clinical practice. A second limitation is that patients were scanned at a single center using a single CT model and manufacturer. As different CT units vary in their geometry, beam filtration, and reconstruction algorithms, the uLDCT scan parameters used in this study cannot be directly transferred to a different CT model. Third, expert chest radiologists read the studies, and the results may be different if studies were reviewed by general radiologists. However, the results of this study are encouraging for other oncology centers to revisit the standard-of-care CT protocol for immunocompromised patients and to lower the effective radiation dose into the sub-mSv range. The fourth limitation is that very subtle findings such as ground-glass opacity might be less visible and under-recognized on uLDCT and this should be taken into account.

To successfully transition the established clinical practice from LDCT to uLDCT, it may be useful to perform LDCT as a baseline study when clinically required in immunocompromised patients and then to use uLDCT for follow-up examinations. This would allow oncologists and radiologists to gain experience and comfort with the diagnostic performance of uLDCT in their patients. The next phase would be to institute uLDCT at baseline and for follow-up in all non-obese patients.

## 6. Conclusions

Ultra-low-dose thoracic computed tomography performed with a 75% radiation dose reduction compared to LDCT has a clinically acceptable image quality and radiologist performance in the detection of major and minor radiological criteria of invasive fungal infection in immunocompromised patients with a BMI < 30.0 kg/m^2^. This protocol should be considered when excluding invasive fungal infection in non-obese immunocompromised patients. It may be pragmatic to initially introduce uLDCT for surveillance imaging only, using LDCT as a baseline, to facilitate a smooth transition prior to implementing uLDCT for baseline and surveillance imaging.

## Figures and Tables

**Table 1:** Likert scores for the evaluation of image quality, diagnostic confidence of invasive fungal infection, detection of major and minor EORTC radiological criteria for fungal disease.

Category	Likert Score
**Image Quality (Anatomy)**	1—Poor 2—Suboptimal 3—Good 4—Very Good 5—Excellent
Airways	
Vasculature	
Lung parenchyma	
Pleura	
Bone	
**Image Quality (Anatomy)**	
Streak artifacts	
Image noise	
Image sharpness	
**Lung Reconstruction Diagnostic Confidence**	1—Unacceptable 2—Limited confidence 3—Average (**clinically acceptable**) 4—Above average 5—Fully confident
**EORTC Criteria**	1—Definitely none 2—Unlikely 3—Not sure 4—Likely 5—Definitely Present
Major: • Macro nodule. • Halo sign. • Cavitation. • Consolidation.	
Minor: • Ground-glass opacity (GGO). • Nodules (4–10 mm), clustered or isolated.	

Abbreviation: EORTC, European Organization for Research and Treatment of Cancer.

**Table 2:** Cohort demographics and dose parameters.

	All	Underweight	Normal	Overweight	Obese
**Number of patients**	100	5	46	28	21
**Male/Female**	53/47	3/2	26/20	16/12	8/13
**Age (years)**	55.3 ± 14.7 [18–80]	51.4 ± 18.6 [22–69]	55.3 ± 15.8 [19–80]	56.3 ± 13.1 [27–74]	55.1 ± 15.0 [18–76]
**BMI ^a^ (kg/m^2^)**	26.2 ± 6.3 [14.7–60.2]	16.5 ± 1.6 [14.7–18.2]	22.6 ± 1.4 [19.8–24.9]	27.1 ± 1.5 [25.1–29.4]	34.0 ± 3.8 [30.0–44.6]
**LDCT DLP ^a^** **(mGy × cm)**	125.7 ± 18.9 [85.5–160.8]	123.8 ± 21.7 [93.9–148.5]	123.2 ± 21.5 [85.5–158.3]	125.2 ± 17.4 [95.7–160.8]	132.0 ± 12.9 [100.6–150.7]
**uLDCT DLP ^a^ (mGy × cm)**	30.5 ± 4.7 [20.6–39.1]	30.0 ± 5.4 [22.7–36.1]	29.9 ± 5.3 [20.6–38.5]	30.4 ± 4.3 [23.1–39.1]	32.1 ± 3.2 [24.3–36.7]
**LDCT Effective ^a^ Dose (mSv)**	1.76 ± 0.26 [1.20–2.25]	1.73 ± 0.30 [1.31–2.08]	1.73 ± 0.30 [1.20–2.22]	1.75 ± 0.24 [1.34–2.25]	1.85 ± 0.18 [1.41–2.11]
**uLDCT Effective ^a^ Dose (mSv)**	0.43 ± 0.07 [0.29–0.55]	0.42 ± 0.08 [0.32–0.51]	0.42 ± 0.07 [0.29–0.54]	0.43 ± 0.06 [0.32–0.55]	0.45 ± 0.04 [0.34–0.51]

Abbreviation: BMI, body mass index; DLP, Dose Length Product; LDCT, LDCT; uLDCT, ultra-LDCT. ^a^ Values are presented as mean ± std [range].

**Table 3:** European Organization for Research and Treatment of Cancer (EORTC) major and minor radiological findings for presence of fungal disease in ultra-LDCT scans. LDCT results are considered ground truth and detection is calculated on a per patient basis.

	True Positive	False Negative	False Positive	True Negative	Sensitivity (%)	Specificity (%)	Accuracy (%)
Any Major Radiological Sign *	**24**	**2**	**2**	**72**	**92.3%**	**97.3%**	**96.0%**
**Macro Nodule**	4	0	0	96	100%	100%	100%
**Halo Sign**	2	1	0	97	66.7	100.0	99.0
**Cavitation**	2	0	0	98	100.0	100.0	100.0
**Consolidation**	22	2	3	73	91.7	96.1	95.0
Any Minor Radiological Sign *	**71**	**13**	**3**	**13**	**84.5%**	**81.3%**	**84.0%**
**Nodules** **(4–10 mm)**	49	13	8	30	79.0	78.9	79.0
**GGO**	56	16	3	25	77.8	89.3	81.0

* Subgroup analyses by removing the obese patient cohort results in sensitivity, specificity, and accuracy values as follows: (1) for any major radiological sign, 90.9%, 96.5%, and 94.9%; (2) for any minor radiological sign, 85.1%, 75.0%, and 83.5%.

**Table 4:** Interclass correlation coefficient and 95% Confidence Interval among the three radiologist readers for European Organization for Research and Treatment of Cancer (EORTC) major and minor radiological findings.

	LDCT ICC ^a^ [95% CI]	uLDCT ICC ^a^ [95% CI]
**EORTC Major Findings**		
**Any major findings**	0.83 [0.45–0.99]	0.82 [0.46–0.97]
**Macro nodule**	0.87 [0.2–0.97]	0.83 [0.27–0.94]
**Halo sign**	0.74 [0.07–0.93]	0.95 [0.29–0.98]
**Cavitation**	0.92 [0.25–0.98]	0.96 [0.27–1]
**Consolidation**	0.84 [0.45–0.97]	0.85 [0.49–0.98]
**EORTC Minor Findings**		
**Any minor findings**	0.65 [0.22–0.93]	0.66 [0.32–0.92]
**GGO**	0.67 [0.33–0.92]	0.69 [0.39–0.94]
**Nodules (4–10 mm)**	0.58 [0.22–0.98]	0.51 [0.25–0.89]

Abbreviation: CI, Confidence Interval, LDCT, LDCT; ICC, interclass correlation coefficient; uLDCT, ultra-LDCT. ^a^ Two-way mixed ANOVA model without interaction (random subject and fixed rater effects) was employed. The analysis was performed for 100 subjects [100 LDCT (LDCT) or 100 ultra-LDCT (uLDCT) scans], 3 raters (3 readers), and 5 possible categories: 1 to 5.

## Data Availability

Study data is unavailable due to privacy or ethical restrictions.
